# Extrapyramidal Symptoms with Administration of Lenalidomide Maintenance Therapy for Multiple Myeloma

**DOI:** 10.7759/cureus.3349

**Published:** 2018-09-24

**Authors:** FNU Sagar, Saad Ullah Malik, Supranee Soontornprueksa, Awais Ijaz, Muhammad Usman, Ali Younas Khan, Pavan Tenneti, Mohammad A Fraz, Faiz Anwer

**Affiliations:** 1 Internal Medicine, University of Arizona, Tucson, USA; 2 Hematology and Oncology, University of Arizona, Tucson, USA; 3 Oncology, University of Arizona, Tucson, USA; 4 Internal Medicine, Banner University Medical Center, Tucson, USA

**Keywords:** lenalidomide, thalidomide, extrapyramidal symptoms, multiple myeloma, tardive dyskinesia

## Abstract

Lenalidomide is commonly used as induction or maintenance therapy in multiple myeloma. We report a case of 71-year-old female presenting with tardive dyskinesia-like symptoms one month after starting her lenalidomide maintenance therapy after high-dose chemotherapy and autologous hematopoietic stem cell rescue.

Her symptoms evolved over days to pronounced uncontrollable limb movements, tongue smacking, lip-smacking, abnormal sounds, and tongue biting. The patient categorically denied any exposure to other drugs which are known to cause symptoms of tardive dyskinesia. The patient underwent a thorough evaluation, stopped the lenalidomide, and received therapy to control her symptoms with a gradual improvement over a six-week period. There is a paucity of literature on the association of lenalidomide with tardive dyskinesia. Common central nervous system-related side effects include peripheral neuropathy, dizziness, dysgeusia, headache, tremor, somnolence, and memory impairment. Very few studies in the existing literature have reported an association of tardive dyskinesia with lenalidomide therapy. Here, we present a case of an elderly female with multiple myeloma who developed severe tardive dyskinesia while she was on lenalidomide maintenance therapy.

## Introduction

Lenalidomide (LEN) is commonly used in the treatment of multiple myeloma (MM) as induction or maintenance therapy following autologous hematopoietic stem cell transplant (Auto-HSCT). It belongs to the class of immunomodulatory drugs (IMiDs) which acts by downregulating transcription factors like Ikaros (IKZF1) and Aiolos (IKZF3). These transcription factors increase production of interferon regulatory factor (IRF4) which, in turn, regulates malignancy-specific gene expression in MM [1]. It functions as an antimyeloma agent by stimulating the proliferation of anti-CD3 (cluster of differentiation 3) stimulated T cells and inhibiting the secretion of proinflammatory cytokines and angiogenic factors in the cells [2-3]. LEN is chemically related to its parent drug, thalidomide, but with a better side effect profile. Adverse effects of LEN may include fatigue, muscle cramps, thrombocytopenia, neutropenia, dizziness, tremors, and neuropathy. Herein, we present a case of possible association between LEN use and tardive dyskinesia (TD).

## Case presentation

A 71-year-old Caucasian female (otherwise asymptomatic) was noted to have persistent pancytopenia since 2015. On January 12, 2017, she underwent multiple myeloma staging workup. Lab findings revealed hemoglobin: 9.9 mg/dl; platelets: 110 x 103/μl; absolute neutrophils: 1.08 x 103/μl; lymphocytes: 1.00 x 103/μl; monocytes: 0.18 x 103/μl; lactate dehydrogenase (LDH): 177 units per liter (U/L); alkaline phosphatase: 129 U/L; calcium: 9.3 mg/dl; and creatinine: 0.8 mg/dl. The serum immunoassay revealed an elevated immunoglobulin A (IgA) of 1,857 mg/dl with a low immunoglobulin M (IgM) of 26 mg/dl and a normal immunoglobulin G (IgG) of 626 mg/dl. There was a monoclonal spike (M-spike) (1.8) of the IgA kappa (IgA-K) type in the serum. The serum level of free kappa light chain was elevated at 350.06 and the kappa/lambda ratio was 30.18. The plasma b-2 microglobulin was high at 3.86. A bone marrow (BM) examination showed 30 - 40% IgA-K monoclonal plasma cells, and she had multiple lytic bone lesions in the skull, pelvis, humerus, and femur. She was diagnosed with MM, Stage II (International Staging System). Fluorescence in situ hybridization (FISH) revealed a loss of 1 p in 94.5% of cells, trisomy of chromosome 7 in 85% of cells, trisomy of chromosome 9 in 76.5% of cells, trisomy of chromosome 11 in 88.5% of cells, gain of 3' immunoglobulin heavy-chain gene (IgH) in 87.0% of cells, along with a gain of 1q21, trisomy of chromosomes 7, 9, and 11, and partial gain of IgH.

She received three cycles of carfilzomib, lenalidomide, and dexamethasone (KRd) for remission induction from January 30 to March 2017 followed by high-dose melphalan and auto-HSCT as consolidation on July 6, 2017. She was started on maintenance LEN (10 mg/day) in October of 2017. Approximately three months after starting LEN maintenance on January 24, 2018, she complained of acute onset jaw pain that evolved into abnormal movements, such as continuous lip-smacking, blinking of eyes, frowning, and chewing movements, which further progressed to uncontrolled verbal tics and difficulty with speech over the next few days. One week after the onset of her symptoms, the LEN maintenance therapy was stopped. Gradually, her symptoms worsened and were managed with lorazepam. She underwent a neurological evaluation, including computerized tomography (CT) scan and magnetic resonance imaging (MRI) of the brain, which was negative. The abnormal involuntary movement scale (AIMS) was used to detect TD and also to follow the severity of TD symptoms over time. The rating of two or higher is diagnostic for TD. Table 1 shows the patient's score on her first neurologist visit.

**Table 1:** Abnormal involuntary movement scale (AIMS)

	Score
Facial and Oral Movements	
Muscles of facial expression, e.g., movements of forehead, eyebrows, periorbital area, cheeks. Include frowning, blinking, grimacing of the upper face.	2.5
Lips and perioral area, e.g., puckering, pouting, smacking.	3
Jaw, e.g., biting, clenching, chewing, mouth opening, lateral movement.	2
Tongue. Rate only increase in movement both in and out of the mouth, not an inability to sustain movement.	4
Extremity Movements	
Upper (arms, wrists, hands, fingers). Include movements that are choreic (rapid, objectively purposeless, irregular, spontaneous) or athetoid (slow, irregular, complex, serpentine). Do not include tremor (repetitive, regular, rhythmic movements).	3
Lower (legs, knees, ankles, toes), e.g., lateral knee movement, foot tapping, heel dropping, foot squirming, and inversion and eversion of the foot.	1
Trunk Movements	
Neck, shoulders, hips, e.g., rocking, twisting, squirming, and pelvic gyrations. Include diaphragmatic movements	2
Global Judgments	
Severity of abnormal movements overall	3
Incapacitation due to abnormal movements.	3
Patient's awareness of abnormal movements.	3
Dental Status	
Current problems with teeth and/or dentures.	No
Does the patient usually wear dentures?	No
Endentia?	No
Do movements disappear with sleep?	No

Her symptoms were managed with diphenhydramine, diazepam, and later with clonazepam. Her home medications included acyclovir, aspirin, dexamethasone, ibuprofen, loratadine, magnesium oxide, and lorazepam. None of these are associated with drug-induced tardive-like dyskinesia/dystonia. The patient continued clonazepam, leading to a gradual improvement in her symptoms. Since January 24, 2018, she has not taken LEN, and her disease is in a remission phase.

## Discussion

**Figure 1:**
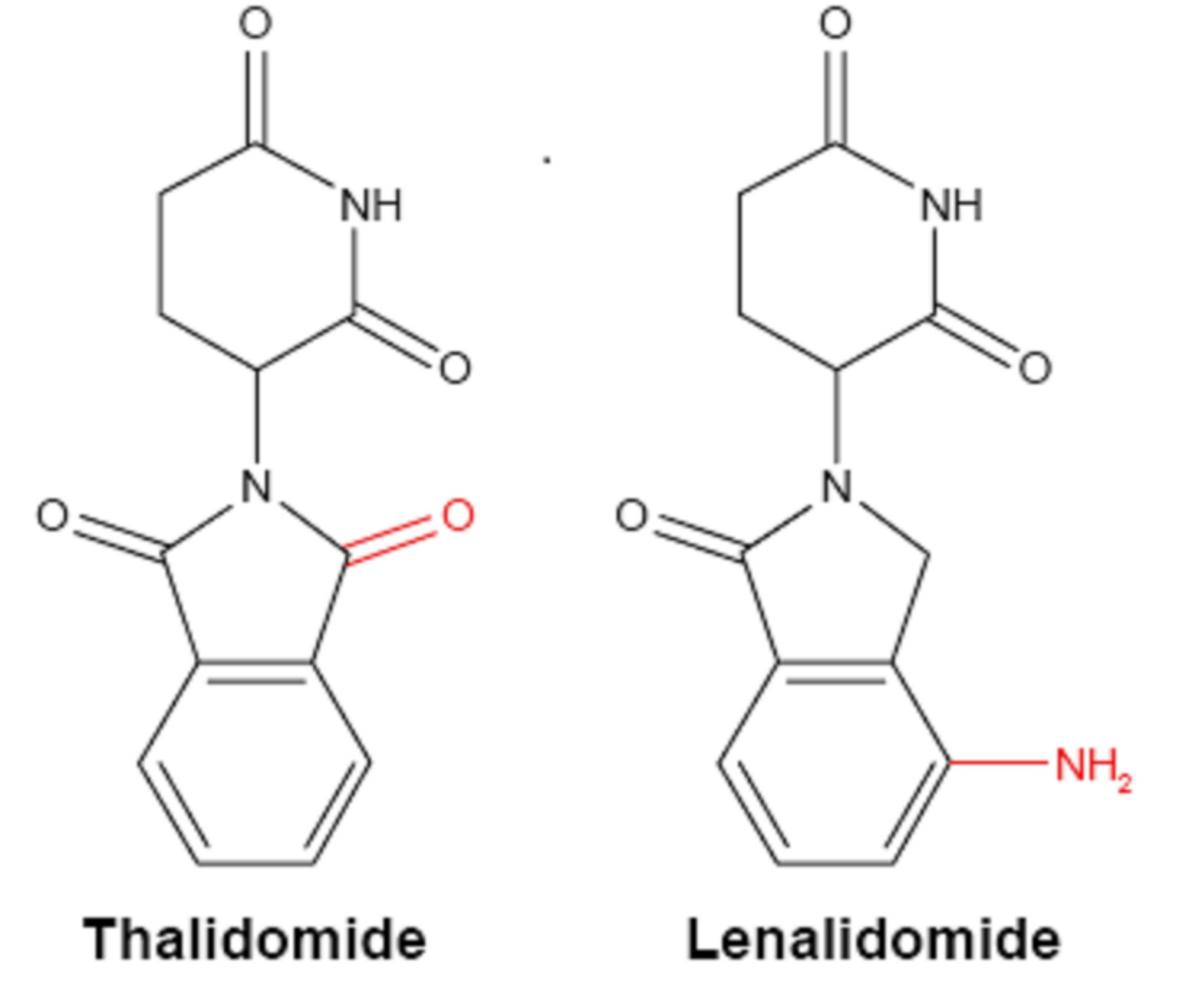
Chemical structure of immunomodulatory drugs Common structural features are shown in black, whereas red indicates the differences between the two structures. O: oxygen; NH: imide group; N: nitrogen, NH2: amino group Modified figure from Ríos-Tamayo et al. [6]

Lenalidomide therapy has significantly improved the response rates and progression-free survival among patients with MM [4]. Lenalidomide is derived from thalidomide but is more potent than its parent drug. The former has an additional amino group at position 4 of the phthaloyl ring and removal of a carbonyl group from the phthaloyl ring (as shown in Figure 1) [5-6]. Theoretically, this similarity in structure may lead to an overlap in toxicities, i.e. neuropathies (occurring in more than 50% of patients on thalidomide) [7-8]. However, the exact pathophysiology leading to central nervous system (CNS) side-effects caused by IMiDs is poorly understood, and there is no proposed mechanism for interaction of lenalidomide with CNS leading to TD. Neurologic side effects that may occur with the use of LEN include headache (1.1%), dizziness (3.4%), tremors (1.1%), peripheral neuropathy (1.7%), and insomnia (1.1%), and very rarely, TD (which is commonly seen with the use of antipsychotics) [9-10]. LEN-associated TD was especially found to be more common in females, 60+ years old, patients taking the drug for < 1 month who also take the medication metoclopramide, and have indigestion. Extrapyramidal symptoms (EPS) have also been reported with the use of thalidomide. In a study by Marchetti et al., 38 patients (60.3%) on thalidomide experienced neurologic side effects and 1.6% patients had extrapyramidal symptoms [11]. Similarly, Crystal et al. reported an acute worsening of Parkinson's disease with the use of thalidomide, which had been given to a patient of myelofibrosis with myeloid metaplasia. His symptoms partly resolved within two weeks of discontinuation of thalidomide [12].

Our patient had no history of past exposure to medications associated with drug-induced TD/dystonia at the time of her symptoms. Neurologic adverse effects, including extrapyramidal side effects, can occur with thalidomide; the structural similarity between these drugs can potentially contribute to similar toxicities and could possibly be the reason for TD caused by LEN in this patient. Further studies are suggested to establish the association of TD with LEN use in MM patients.

## Conclusions

LEN is a medication commonly used during induction and maintenance therapy of MM that significantly improves the progression-free survival and overall survival in these patients. Neurologic side effects commonly associated with LEN therapy are peripheral neuropathy, dizziness, dysgeusia, headache, tremor, somnolence, and memory impairment; however, it may cause TD rarely. TD can be socially and physically disabling for the patient, causing impairment of mobility, speech, and interference with eating. This makes it imperative to report these rare and unusual side effects for prompt management. Patient education is also important for early detection and treatment. Further studies should be carried out to establish the association of LEN with the development of TD focusing on how the structural similarity between the LEN and thalidomide can potentially contribute to such toxicities.
